# Tumor Inflammation, Obesity, and Proliferative Status as Biomarkers in Gastroesophageal Adenocarcinoma

**DOI:** 10.3390/jpm11121324

**Published:** 2021-12-07

**Authors:** Sarbajit Mukherjee, R. J. Seager, Yong Hee Lee, Jeffrey M. Conroy, Pawel Kalinski, Sarabjot Pabla

**Affiliations:** 1 Department of Medicine, Roswell Park Comprehensive Cancer Center, Elm and Carlton Streets, New York, NY 14206, USA; 2 Bioinformatics, OmniSeq, Inc., 700 Ellicott Street, Buffalo, NY 14203, USA; robert.seager@omniseq.com (R.J.S.); yonghee.lee@omniseq.com (Y.H.L.); sarabjot.pabla@omniseq.com (S.P.); 3 Roswell Park Comprehensive Cancer Center, Center for Personalized Medicine, Elm and Carlton Streets, New York, NY 14206, USA; jeffrey.conroy@omniseq.com; 4 Research and Development, OmniSeq, Inc., 700 Ellicott Street, Buffalo, NY 14203, USA; 5 Department of Immunology, Roswell Park Comprehensive Cancer Center, Elm and Carlton Streets, New York, NY 14206, USA; pawel.kalinski@roswellpark.org

**Keywords:** biomarker, gastric cancer, esophageal cancer, immunotherapy, obesity, inflammation, proliferation, survival, BMI, body mass index

## Abstract

Recent epidemiological studies have shown that obesity, typically measured by increased body mass index (BMI), is associated with an increased risk of gastroesophageal adenocarcinoma (GEAC), but the contributing molecular and immune mechanisms remain unknown. Since obesity is known to promote chronic inflammation, we hypothesized that obesity leads to inflammation-related immune dysfunction, which can be reversed by immune-modulating therapy. To test our hypothesis, we examined the clinical and molecular data from advanced GEAC patients. To this end, 46 GEAC tumors were evaluated for biomarkers representing tumor inflammation, cell proliferation, and PD-L1 expression. A CoxPH regression model with potential co-variates, followed by pairwise post hoc analysis, revealed that inflammation in the GEAC tumor microenvironment is associated with improved overall survival, regardless of BMI. We also observed a significant association between cell proliferation and progression-free survival in overweight individuals who received immune-modulating therapy. In conclusion, our data confirm the role of the immune system in the natural course of GEAC and its responses to immunotherapies, but do not support the role of BMI as an independent clinically relevant biomarker in this group of patients.

## 1. Introduction

Gastroesophageal cancers, combined, are the most prevalent type of gastrointestinal cancers worldwide. Esophageal cancer is a global health problem, with an estimated annual incidence of 455,800 and 400,200 annual deaths in 2012 [1]. In the US, most esophageal cancers are adenocarcinoma, usually confined to the distal esophagus [2]. On the other hand, the incidence of gastroesophageal junction adenocarcinoma has also steadily increased in the US since 1970 [2]. Gastric cancer is responsible for approximately 952,000 new diagnoses worldwide (6.8% of new cancer cases worldwide) and 723,000 deaths annually (8.8% of the total) [3]. Recently, both nivolumab and pembrolizumab have been approved for use in the first-line setting, combined with chemotherapy, in metastatic or unresectable gastroesophageal cancer patients. However, their benefit seems to be apparent in a limited group of patients. This outcome profile, combined with potentially severe immune-related adverse effects (irAEs) and very high costs, means a reliable predictive biomarker is urgently needed. Recently, the role of inflammation in the tumor microenvironment (TME) is being evaluated as a biomarker of the response to immune checkpoint inhibitors (ICIs) [4]. Additionally, inflammation in the TME also seems to modulate the response to trastuzumab and ramucirumab, two commonly used drugs in advanced GEAC, with known immune-modulating properties [5,6].

Obesity is a major risk factor for the development of several types of cancers, including GEAC [7]. Obesity is generally known to cause a chronic inflammatory state, leading to immune dysfunction and the development of cancers [8]. We recently showed that obesity enhances PD-1-mediated T-cell dysfunction at least partly by leptin signaling [9], but, interestingly, obese patients respond better to treatment with immune checkpoint inhibitors. Based on this preliminary data, we hypothesized that obesity creates inflammation-mediated immune dysfunction in the tumor microenvironment (TME), and this can be reversed by immune-modulating agents, leading to an improved outcome. To test our hypothesis, we obtained the clinical and RNA-seq data from advanced GEAC patients and further analyzed the gene expression data to delineate associations between obesity, inflammation in the TME, and clinical outcomes.

## 2. Materials and Methods

### 2.1. Patients and Clinical Data

Samples from 46 GEAC patients were included in this study. Based on similar molecular features and clinical outcomes in prior research studies, we decided to analyze esophageal, gastroesophageal junction and gastric adenocarcinoma together [10,11,12]. We used the Siewart classification for defining gastroesophageal junction cancer [13]. We used the WHO classification to define overweight patients. A BMI cutoff of 25 or over was defined as overweight in our study [14].

### 2.2. Quality Assessment of Clinical FFPE Tissue Specimens

FFPE blocks were cut into 5 μm sections onto positively charged slides. One section from each block was stained with H&E and assessed by a board-certified anatomical pathologist for tumor representation adequacy, tissue preservation quality, signs of necrosis, and fixation or handling issues. Any specimens with <5% tumor tissue content or >50% necrosis were excluded from analysis. In most cases, with or without tumor microdissection, tissue from 3–5 unstained slide sections was required to meet the assay RNA requirements (10 ng).

### 2.3. Immunohistochemical Studies

Regardless of tumor type, PD-L1 expression on the surface of cancer cells was assessed in all cases by Dako PD-L1 IHC 22C3 pharmDx (Agilent, Santa Clara, CA, 90001, USA). A board-certified anatomical pathologist scored PD-L1 expression levels according to published guidelines [15], where a tumor proportion score (TPS) greater than 1% was deemed a positive result (PD-L1+) and a TSP less than 1% was deemed a negative result (PD-L1−).

### 2.4. Nucleic Acid Isolation and Gene Expression

RNA was extracted from each sample and gene expression was assessed by RNA-seq, as previously described [16]. RNA (RiboGreen staining) was quantified by Qubit fluorometer (Thermo Fisher Scientific, Waltham, MA, 02188, USA). Gene expression was assessed by RNA sequencing of 395 transcripts on samples meeting validated quality control (QC) thresholds [16]. RNA libraries were sequenced to appropriate depth on the Ion Torrent S5XL sequencer (Thermo Fisher Scientific, Waltham, MA, 02188, USA).

### 2.5. Data Analyses

RNA-seq absolute reads for each transcript were generated using the Torrent Suite plugin immuneResponseRNA (Thermo Fisher Scientific, Waltham, MA, 02188, USA) [16]. For each transcript, the library preparation background was the absolute read count from the non-transcript control (NTC). This background value was subtracted from the absolute read counts for the same transcript in all other samples of the same batch. To enable comparisons of NGS measurements between runs, normalized reads per million (nRPM) were generated from each background-subtracted read count by comparing each housekeeping (HK) gene background-subtracted read against a previously determined HK RPM profile. For each gene, gene expression ranks were calculated by converting nRPM expression values to a percentile rank between 0 and 100, compared against a reference population of 735 solid tumors spanning 35 histologies [16].

Unsupervised hierarchical clustering with Pearson’s correlation (R) as a measure of distance was initially used to visualize the overall gene expression landscape of the patient cohort, as previously described [17]. K-means clustering was then used to refine these results, generating the following 3 stable clusters of genes: cancer testis antigen (CTA) genes, genes associated with inflammation, and other cell proliferation and neoplasm genes. K-means clustering was applied in a similar manner to generate three stable clusters of patients, which were designated as hot, moderate, and cold tumors, based on the expression levels of genes in the aforementioned inflammation-associated gene cluster, with hot tumors having the highest expression levels and cold tumors having the lowest expression levels of these inflammation-associated genes.

A cell proliferation signature was generated for each patient by calculating the mean expression of 10 cell proliferation-related genes, including MKI-67. This cell proliferation signature has been previously published to describe the tumor cell proliferation state where proliferation signal from whole tissue is counted (tumor and immune cells) [18,19].

Additionally, we performed survival analyses, generating Kaplan–Meier survival curves and comparing them using a log-rank test. Further, we used a Cox proportional hazards model to compute hazard ratios (HRs), 95% confidence intervals (CIs), and *p* values for testing the effect of co-variates (cell proliferation, BMI, inflammation status) on overall survival.

## 3. Results

### 3.1. Patient Cohort

**Figure 1:**
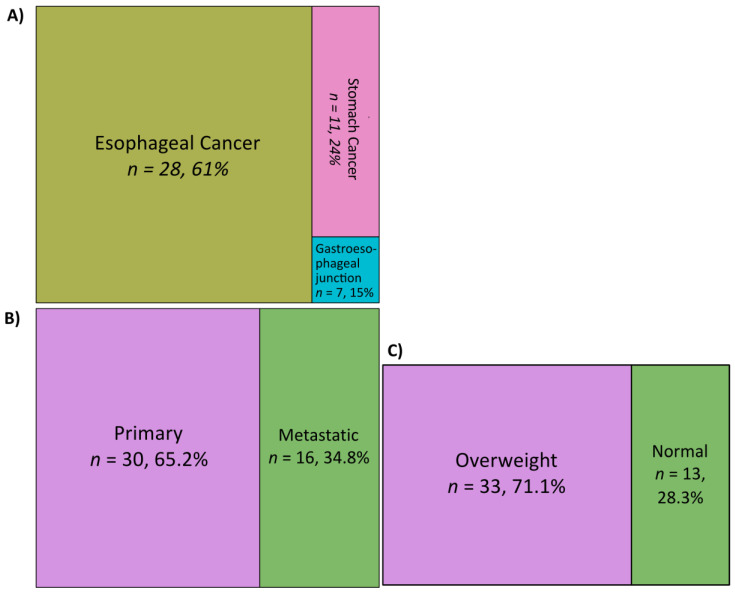
Patient cohort composition by (**A**) cancer type, (**B**) PMR status, and (**C**) BMI status.

Of the 46 GEAC patients comprising the study cohort, 61% of these patients had esophageal cancer, 24% had stomach cancer, and 15% had cancer of the gastroesophageal junction (Figure 1). Tissue was obtained from primary tumors in 65.2% of the patients, while the remainder were metastatic (Figure 1). The median age at diagnosis was 64 years, and the cohort was 89.1% male and 10.9% female (Table 1). Half of the patients had received immune-modulating drugs (nivolumab, pembrolizumab, ramucirumab, or trastuzumab), while the remainder had not (Table 1). Further, 71.1% of the patients were overweight (BMI ≥ 25), while the remainder were in the normal BMI range (BMI < 25) (Figure 1).

### 3.2. Unsupervised Gene Expression Analysis

**Figure 2:**
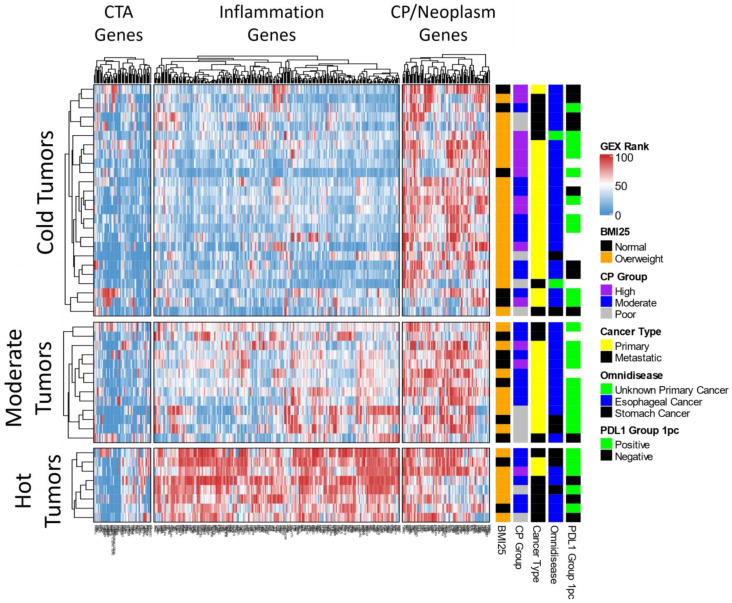
Unsupervised clustering of 395 genes (columns) across 46 samples (rows) leading to discovery of hot, moderate and cold tumor subtypes. Annotated for each sample are BMI, cell proliferation status, cancer type, disease type (omnidisease), and PDL1 (positive: ≥1%).

Unsupervised analysis of the gene expression data revealed three distinct patient clusters, namely, hot, moderate, and cold (Figure 2). As expected, hot tumors were over-represented by >250 immune-related genes (inflammation gene cluster), and 6/8 (75%) of the patients in this cluster were overweight. On the other hand, cold tumors were over-represented by high cell proliferation and overexpression of cancer testis antigen genes, and 20/25 (80%) of the cases in this cluster were overweight. Across the entire cohort of 46 GEAC patients, the majority of cases were cold tumors (52.2%) and the minority of cases were hot tumors (17.4%; Table 1). Additionally, a plurality of cases were moderately proliferative (45.7%), while the minority were poorly proliferative (23.9%; Table 1).

### 3.3. BMI Status and Biomarkers

**Figure 3:**
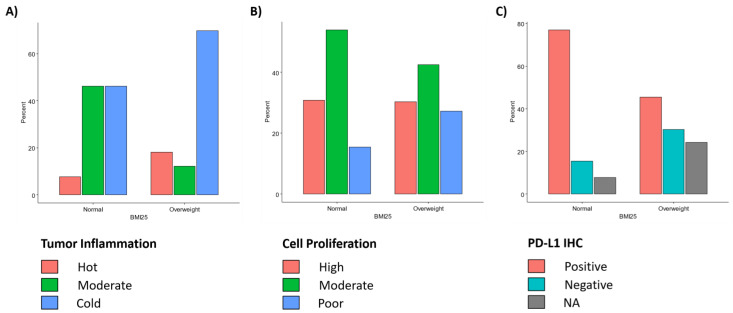
Proportions of (**A**) inflammation, (**B**) cell proliferation and (**C**) PD-L1 IHC biomarker in two BMI groups.

When examining the proportions of each BMI group represented by each tumor inflammation group, we found that normal-weight cases have a significantly higher proportion of moderately inflamed tumors, compared to overweight cases (*p* = 0.03338; Figure 3a). By analyzing the proportions of each BMI group represented by each cell proliferation group in a similar way, we determined that cell proliferation was not significantly different between the two BMI groups (Figure 3b). Finally, by analyzing the proportions of each BMI group represented by each PD-L1 IHC group, we found that PD-L1 showed a trend towards higher expression in normal-BMI patients than in high-BMI patents, although this difference did not reach statistical significance (*p* = 0.109; Figure 3c).

### 3.4. Overall Survival, BMI and Other Biomarkers

To investigate the prognostic power of BMI and gene expression biomarkers, we performed Kaplan–Meier analysis of the complete cohort (Table 2). We found that hot tumors had significantly improved OS (orange shaded values) compared to moderate (*p* < 0.041) or cold (*p* = 0.01), with gender identified as a significant co-variate. Additionally, tumor inflammation status was still significant (green shaded values) when BMI and cell proliferation biomarkers were accounted for in a Cox regression analysis.

We performed an additional Kaplan–Meier survival analysis, looking specifically at overall survival and progression-free survival in patients treated with immune-modulating drugs (IMD), including, but not limited to, immune checkpoint inhibitors (nivolumab, pembrolizumab, ramucirumab, or trastuzumab) (Table 3). Comparing survival between the inflammation groups, we found that the hot status showed a trend towards improved progression-free survival in IMD-treated cases when compared to moderate (*p* = 0.190) or cold cases (*p* = 0.174). Additionally, we determined that BMI status and cell proliferation were not significantly associated with OS benefit or improved PFS (data not shown).

To further investigate the discriminatory power of inflammation and cell proliferation on separating PFS in IMD-treated BMI groups, we performed interaction analysis (Table 4). Confirming the finding above, this analysis revealed that in overweight cases that were moderately proliferative, there was significantly decreased PFS for cold vs. hot tumors (HR = 31.129; CI = 2.117–457.83).

## 4. Discussion

In obese patients, several factors work together to result in T-cell dysfunction, ultimately leading to an exhausted T-cell phenotype [20]. Our previous work in a metastatic GEAC population showed that overweight individuals (BMI ≥ 25) were more likely to express PD-L1 in their TME than normal-weight individuals [21]. In addition, many other immune suppressive markers, such as FOXP3 and IDO1, were found to be upregulated in the obese group. Since obesity is well known to cause inflammation, we hypothesized that obesity-induced inflammation leads to immune dysfunction in GEAC. We further sought to investigate if obesity and inflammation in the TME have any prognostic role in metastatic GEAC patients. Unsupervised clustering analysis revealed a set of more than 250 immune response genes in hot tumors that were over-represented by T and B cell activation, as well as interferon gamma pathway genes. A similar RNA-seq-based analysis has revealed the predictive and prognostic utility of NGS-based inflammation signatures in many solid tumors [17]. In this study, we describe the novel application of such inflammation signatures in the context of BMI in a GEAC cohort. Finally, in an exploratory analysis, we looked at the prognostic influence of cell proliferation markers in this patient population. As expected, cell proliferation alone does not predict a response or improved survival to checkpoint inhibition [19]. However, the integration of cell proliferation with inflammation and other traditional biomarkers discriminates overall survival in multiple tumor types [17]. Interestingly, we observed that moderately proliferative cold tumors had significantly worse PFS compared to hot tumors that were moderately proliferative, hinting at a potentially balanced tumor immune microenvironment that can leverage sufficiently proliferated immune components within hot tumors.

Our results indicate that the inflammation in the TME is associated with survival outcomes in metastatic GEAC patients, regardless of obesity. In our patient population, “hot” tumors were associated with improved OS, compared to “cold” or immune-suppressed tumors, with gender identified as a significant co-variate. Gender is known to affect outcomes in several cancers, including GEAC. Our previous research has shown that female patients with gastric cancer had better survival compared to their male counterparts [14]. Furthermore, gender seems to impact the response to cancer immunotherapy [15].

The strength of our study lies in the use of a novel bioinformatics approach to categorize tumors into simple and clinically relevant groups using the immune gene expression profile. Similarly to the previously published data, we showed that inflammation in the TME is associated with survival outcomes in GEAC [16]. Furthermore, our findings seem to suggest that inflammation in the TME could predict a benefit of immune-modulating anti-neoplastic therapy, pending validation in larger cohorts. Our findings are in agreement with the biomarker analysis of the Keynote-059 study, where an association was observed between a novel 18-gene T-cell-inflamed gene expression profiling score and response to pembrolizumab in advanced, previously treated gastric and gastroesophageal junction cancer patients [17].

It should be noted that we defined obesity by BMI. However, BMI is being criticized lately as a measure of obesity, since it cannot differentiate between fat and lean body mass; therefore, investigators are using novel approaches, including imaging techniques, to measure obesity [18]. Additionally, the relatively small number of patients and retrospective nature of the study make it difficult to reach a definite conclusion. Nevertheless, our findings are hypothesis generating and need to be validated in larger, prospective cohorts.

## 5. Conclusions

In summary, we report a comprehensive tumor inflammation landscape that describes the host immune response of GEAC tumors, regardless of BMI status. We further integrate biomarkers of response (PD-L1 IHC) and resistance (cell proliferation) to immune checkpoint inhibition for a more comprehensive understanding of tumor immunity. Tumor inflammation status, alone and in conjunction with other biomarkers, is a potential prognostic biomarker in GEAC. More importantly, this study integrates multiple NGS-based biomarkers to fill the gaps in our understanding of tumor immunity of GEAC. Similar integrative approaches can be used to accelerate drug development pipelines, inform clinical trial selection, and assist routine clinical care of GEAC patients.

## Figures and Tables

**Table 1:** Clinical characteristics of the retrospective cohort.

Variable	Category	Median/Freq
Dx Age	Med (SD)	64 (10.8) years
Sex	Female N (%)	5 (10.9%)
	Male N (%)	41 (89.1%)
Inflammation status	Cold N (%)	25 (54.3%)
	Moderate N (%)	13 (38.3%)
	Hot N (%)	8 (17.4%)
BMI group	Normal N (%)	13 (28.3%)
	Overweight N (%)	33 (71.7%)
Cell Proliferation	High N (%)	14 (30.4%)
	Moderate N (%)	21 (45.7%)
	Poor N (%)	11 (23.9%)
Immune-modulating drugs	Yes N (%)	23 (50%)
	No N (%)	23 (50%)

**Table 2:** OS in all patients.

		OS (Dx Date to Last Follow-Up/Death) *n* = 35		
Model	Comparison	Hazard Ratio	95% CI	*p* Value
Inflammation Status	Cold vs. Hot	24.50	2.40–249.37	<0.01
	Moderate vs. Hot	11.41	1.11–117.37	0.041
	Cold vs. Moderate	2.146	0.44–10.58	0.348
BMI	Overweight vs. Normal	0.73	0.19–2.88	0.653
Cell Proliferation	High vs. Poor	1.18	0.16–8.58	0.871
	Moderate vs. Poor	1.32	0.23–7.44	0.754
	Moderate vs. High	1.12	0.21–5.86	0.894
Inflammation Status + BMI + Cell Proliferation	Cold vs. Hot	49.16	3.67–658.10	<0.01
	Moderate vs. Hot	24.50	1.61–373.30	0.02
	Cold vs. Moderate	2.01	0.35–11.40	0.432
	Overweight vs Normal (BMI_Group)	1.72	0.34–8.60	0.511
	High vs. Poor	0.94	0.09–9.58	0.960
	Moderate vs. Poor	2.40	0.33–17.42	0.386
	Moderate vs. High	2.54	0.40–16.30	0.324

**Table 3:** OS and PFS in IMD-treated patients.

		OS (*n* = 23)			PFS (*n* = 23)		
Model	Comparison	Hazard Ratio	95% CI	*p* Value	Hazard Ratio	95% CI	*p* Value
Inflammation Status	Cold vs. Hot	14,003,350.00	NA	0.994	4.32	0.53–35.48	0.174
	Moderate vs. Hot	11,292,320.00	NA	0.936	4.31	0.49–38.14	0.190
	Cold vs. Moderate	1.24	0.36–4.29	0.734	1	0.31–3.27	0.997

**Table 4:** PFS in interaction model for inflammation groups.

Inflammation Comparison	BMI	Cell Proliferation	Hazard Ratio	CI (Lower)	CI (Upper)
Cold vs. Hot	Overweight	Moderate	31.129	2.117	457.83

## Data Availability

The datasets generated and/or analyzed during the current study are not publicly available due to a non-provisional patent filing covering the methods used to analyze such datasets, but are available from the corresponding author on reasonable request.
